# Chemical, Microbiological and Sensory Stability of Steam Extracted Jaboticaba (*Myrciaria jaboticaba*) Juice

**DOI:** 10.3390/foods10040732

**Published:** 2021-03-30

**Authors:** Ana Beatriz Neves Martins, Mariana Canto, Daniel Perrone, Mariana Monteiro

**Affiliations:** 1 Laboratório de Alimentos Funcionais, Nutrition Institute, Federal University of Rio de Janeiro, Av. Carlos Chagas Filho, 373, CCS, Bloco J, 2° Andar, Sala 16, Rio de Janeiro 21941-902, Brazil; abenmartins@gmail.com; 2 Laboratório de Bioquímica Nutricional e de Alimentos, Chemistry Institute, Federal University of Rio de Janeiro, Av. Athos da Silveira Ramos 149, CT, Bloco A, Sala 528A, Rio de Janeiro 21941-909, Brazil; marianacanto@eq.ufrj.br (M.C.); danielperrone@iq.ufrj.br (D.P.)

**Keywords:** anthocyanins, ellagic acid, gallic acid, Jaboticaba, kinetic models, shelf life, sensory analysis

## Abstract

Jaboticaba (*Myrciaria jaboticaba*) is a Brazilian berry rich in phenolic compounds, much appreciated for its sweet and slightly acid taste, and highly perishable. Thus, we aimed at producing jaboticaba juice by steam extraction and at investigating its microbiological, sensorial and chemical qualities during storage for up to 168 days. Juice was microbiologically safe and even though unsweetened juice was well accepted, sucrose addition further improved flavor (21%), overall impression (11%) and purchase intent (21%) scores. Cyanidin-3-*O*-glucoside (C3G) was the major phenolic (40%), followed by gallic (28%) and ellagic acids (21%). Total phenolics contents decreased from 27% (50 °C) to 50% (25 °C), mainly driven by C3G degradation. At 60 °C, total phenolics contents did not change after 42 days since C3G degradation was counterbalanced by gallic acid formation (129%), which followed zero-order reaction kinetics. Anthocyanins degradation followed first-order reaction kinetics (C3G half-life at 25 °C = 21.7 days) and was associated with color changes during storage. In conclusion, steam extraction followed by hot-filling technique ensured a juice with at least six months of shelf life.

## 1. Introduction

Jaboticaba (or jabuticaba) is a spherical, purple to black colored fruit native to Brazil. It belongs to the *Myrtaceae* family and *Plinia* genus, also referred as *Myrciaria* [1], especially in the scientific field. Its pulp is whitish, juicy and gelatinous, with sweet and slightly acid taste. Beyond its sensory characteristics, the fruit not only shows a valuable nutritional profile—dietary source of carbohydrates, fibers, vitamins and minerals—but also high phenolics content, mainly anthocyanins and ellagitannins. However, as a result of its high perishability, jaboticaba consumption and commercialization are limited to the areas of cultivation and their surroundings [2].

In this sense, the development of jaboticaba-based products is an alternative to prevent post-harvest losses and also a way to add value to the fruit. There is a growing research interest related to the development of jaboticaba-based products, such as juices [3,4], alcoholic beverages [5,6,7], and vinegar [8]. In fact, some products containing jaboticaba, such as yogurts, ice pops, jams, beverages and chocolate have already reached the market, but their availability is still low. Among these products, juices show high commercial potential, as this is an expanding market, and novel fruit drinks, of exotic flavors and with functional properties, are becoming increasingly popular. Moreover, due to consumers concerns related to food quality, the food industry has been focusing on product development based on healthier ingredients and innovative processing technologies [9].

Mechanical extraction methods, such as pressing, squeezing and depulping, are commonly used by the food industry to produce fruit juice, requiring post-production processing to avoid microbial spoilage and chemical changes. As an alternative, steam extraction is a method based on raising water vapor, which reaches the fruit, transferring heat and leaching out the pulp. It is reported to inactivate enzymes and pasteurize juice during its extraction, thus not requiring the application of any further preservation technology [10]. Since it is considered a rapid and low-cost method, small- and medium-scale industries conventionally use steam extraction to produce red grape juice, although juices produced by this technology are slightly diluted due to the transfer of exogenous water during the process [11].

Maintaining juice quality throughout food chain is a relevant issue as quality losses are expected over time. In berries juices, the major challenge is dealing with changes in phenolic compounds, especially anthocyanins, due to their instability towards external factors, such as light, oxygen, temperature [12]. Therefore, the objectives of the present study were to produce jaboticaba juice by steam extraction and to investigate its quality during storage, with emphasis on the changes of its phenolics profile.

## 2. Materials and Methods

### 2.1. Standards and Chemicals

Sugars, organic acids and non-anthocyanins phenolic compounds standards were purchased from Sigma-Aldrich Chemical Co. (St. Louis, MO, USA). Anthocyanins standards were purchased from Indofine Chemical Co. (Hillsborough, NJ, USA). Solvents and water (Milli-Q system, Millipore, Bedford, MA, USA) were HPLC grade.

### 2.2. Jaboticaba Juice Processing and Storage

Jaboticaba fruits (*Myrciaria jaboticaba*, cv. Sabará) from Minas Gerais, Brazil, were purchased at Rio de Janeiro’s agricultural trading center and frozen until use. Unsweetened and sweetened jaboticaba juices (UJJ and SJJ, respectively) were produced by steam extraction using a laboratory-scale stainless-steel steam juicer (Cook N Home, City of Industry, CA) (Figure S1). Thawed and crushed fruits (4 kg) were added after water (3 L) began to boil and extraction was performed for 30 min, yielding approximately 2.2 L of juice. Sucrose (134 g) was added together with the fruits. Juices were produced in triplicate, bottled while hot (~80 °C) in dark glass bottles and stored at 25, 40, 50 and 60 °C in a temperature-controlled oven for up to 168 days to evaluate the impact of long-term storage on juice chemical composition and microbiological and sensory qualities.

The extraction time was chosen after preliminary tests with different extraction times (up to 60 min), taking into account juice yield, anthocyanin content and residual bitter taste. Extraction times superior to 30 min led to less expressive increases in both yield and anthocyanin content while resulted in more noticeable aftertaste. The amount of added sucrose (6% *w/v*) was based on previous literature data [3]. SJJ was produced to evaluate the impact of sucrose addition on jaboticaba juice processing and on its phenolic compounds stability during storage.

### 2.3. Sugar Analysis by HPLC-ELSD

Sugar contents were determined for UJJ stored at 25 °C for up to 112 days, with 56-days intervals. Juices were diluted with acetonitrile and centrifuged (11,300 rpm, 10 min). The supernatant was collected and filtered through a 0.45 μm cellulose ester membrane (Millipore, Barueri, Brazil).

The liquid chromatography system (Shimadzu, Kyoto, Japan) included a LC-20AT quaternary pump, a 8125 manual injector (Rheodyne) with a 20 μL loop, an ELSD-LT II evaporative light scattering detector, a CBM-20A system controller and a DGU-20A5 degasser. Chromatographic separation was performed according to Ma et al. [13], with slight modifications. A normal phase column (NH_2_, 5 μm, 250 mm × 4.6 mm, Zorbax) was used and the mobile phase (isocratic elution) consisted of a mixture of acetonitrile/water (85:15, *v/v*), with a flow rate of 1.0 mL/min. ELSD was set to gain 4, 40 °C and 350 kPa nebulizer gas pressure (N_2_). Identification was performed by comparison with retention time of the respective standard and quantification by external standardization. Limits of detection (LOD) and quantification (LOQ) for sugars were equal to or lower than 0.047 g/100 mL and 1.142 g/100 mL. Data were acquired by LC solution software (Shimadzu Corporation, version 1.25).

### 2.4. Organic Acids Analysis by HPLC-DAD

Organic acids contents were determined for UJJ stored at 25 °C for up to 112 days, with 56-days intervals. Juices were centrifuged and filtered as previously described.

The liquid chromatography system (Shimadzu) included two parallel pumps LC-20AD, automatic injector SIL-20AHT, diode array detector (DAD) SPD-M20A, system controller CBM-20A and degasser DGU-20A5. Chromatographic separation was performed according to Scherer et al. [14], with slight modifications. A reverse phase column (C18, 5 μm, 150 mm × 4.6 mm, Phenomenex) was used and the mobile phase (isocratic elution) consisted of a potassium phosphate buffer solution (KH_2_PO_4_) 0.01 M, pH 2.6 (adjusted with orthophosphoric acid, H_3_PO_4_), with a flow rate of 0.5 mL/min. Identification was performed by comparison with retention time and absorption spectrum of the respective standard. Quantification was performed by external standardization. LOD and LOQ for organic acids were equal to or lower than 0.12 mg/100 mL and 0.36 mg/100 mL. Data were acquired by Lab Solutions software (Shimadzu Corporation, version 5.82).

### 2.5. Phenolic Compounds Analysis by HPLC-DAD-MS

Phenolics contents were determined for both UJJ and SJJ stored at 25 °C for up to 112 days, with 14-days intervals, and at 40, 50 and 60 °C for up to 56 days, with 7-days intervals. Juices were centrifuged and filtered as previously described.

The liquid chromatography system used was the same described in item 2.4, interfaced with a LCMS-2020 mass spectrometer (Shimadzu). Chromatographic separation of anthocyanins was performed according to Inada et al. [2], with slight modifications. A reverse phase column (C18, 5 μm, 150 mm × 4.6 mm, Phenomenex) was used and the mobile phase consisted of a gradient of 1% formic acid and 2% acetonitrile in water (eluent A) and 1% formic acid and 2% acetonitrile in methanol (eluent B), with a flow rate of 1.0 mL/min. Prior to the injection, the column was equilibrated with 23% B. After injection, solvent composition was kept constant until 1 min, increased to 29% B in 2 min, to 33% B in 4 min, to 48% B in 6 min, to 85% B in 8 min and to 95% B in 10 min. Then, it decreased to 23% B in 11 min. Between injections, 10 min intervals were used to re-equilibrate the column with 23% B. Chromatographic separation of non-anthocyanins phenolic compounds was performed according to Inada et al. [2], with slight modifications. A reverse phase column (C18, 5 μm, 250 mm × 4.6 mm, Kromasil) was used and the mobile phase consisted of a gradient of 0.3% formic acid and 1% acetonitrile in water (eluent A) and 1% acetonitrile in methanol (eluent B), with a flow rate of 1.0 mL/min. Prior to the injection, the column was equilibrated with 18.2% B. After injection, solvent composition was increased to 20.2% B in 1 min, to 43.4% B in 18 min, to 85.9% in 23 min and kept constant until 30 min. Between injections, 10 min intervals were used to re-equilibrate the column with 18.2% B.

Electrospray ionization was operated in either negative or positive modes. MS operation conditions were as follows: detector voltage, 3.0 kV; interface temperature, 350 °C; desolvation line temperature, 250 °C; nebulizing gas (ultra-pure N_2_) flow, 1.5 L/min; heat block, 200 °C; drying gas (ultra-pure N_2_) flow, 15 L/min. Identification was performed by comparison with retention time, absorption spectra and m/z of the ions of the respective standard. Quantification was performed by external standardization using DAD signal. LOD and LOQ for phenolic compounds were equal to or lower than 0.006 mg/100 mL and 0.017 mg/100 mL. Data were acquired by Lab solutions software.

### 2.6. Instrumental Color

Instrumental color was evaluated using the CIELab color space (Konica Minolta colorimeter CR-400, Tokyo, Japan) for both UJJ and SJJ stored at 25 °C for up to 112 days, with 14-days intervals, and at 40, 50 and 60 °C for up to 56 days, with 7-days intervals.

Total color difference (ΔE*) during storage, between the initial storage time (*t*_0_) and any given time (*t_i_*), was calculated using the following equation:

$$\Delta E^{*}\;=\;\sqrt{{\left(L_{t_{0}}^{*}-L_{t_{i}}^{*}\right)}^{2}+{\left(a_{t_{0}}^{*}-a_{t_{i}}^{*}\right)}^{2}+{\left(b_{t_{0}}^{*}-b_{t_{i}}^{*}\right)}^{2}}$$

### 2.7. Kinetics of Degradation and Formation of Phenolic Compounds

Kinetic models were fitted to the order of reaction of gallic acid formation and anthocyanins degradation, zero and first-order, respectively. The following general expression (Equation (1)) was used to determine the reaction rate

(1) $$-d\left[C\right]/dt\;=\;k\;{\left[C\right]}^{n}$$

where [*C*] is the concentration of the phenolic compounds under consideration, *t* the reaction time, *k* the rate constant and *n* the order of the reaction. As a result, zero (*n* = 0) and first-order (*n* = 1) reaction rates, at constant temperature, are expressed as Equation (2) and Equation (3), respectively, after integration and logarithmic transformation [15].

(2) $$\;{\left[C\right]}_{t}\;=\;{\left[C\right]}_{0}-kt$$

(3) $$\ln\;{\left[C\right]}_{t}\;=\;\ln\;{\left[C\right]}_{0}-kt$$

Zero and first-order rate constants (*k*) at each temperature were determined through linear regression (*k* = −slope), by plotting [*C*] against time and ln ([*C*]*_t_*/[*C*]_0_) against time, respectively.

The half-life time (*t*_1/2_), which is the time required for anthocyanins to degrade to 50% of their initial contents at given temperatures, was calculated from the rate constant *k*, as *t*_1/2_ = ln 2/*k*.

The activation energy (*E_a_*; kJ mol^−1^) of the reactions were calculated from rate constants (*k*) values observed at each experimental temperature, using the Arrhenius equation (Equation (4))

(4) $$k\;=\;A\;e^{\left(-E_{a}/RT\right)}$$

where *A* is the frequency factor (day^−1^), *R* the universal gas constant (8.3145 J mol^−1^ K^−1^) and *T* the absolute temperature (K) [15].

### 2.8. Microbiological Analyses

Microbiological analyses of UJJ were performed according to Downes and Ito [16] investigating the absence of thermotolerant coliforms bacteria, *Salmonella* spp., heterotrophic bacteria, lactic bacteria, yeasts and molds, every 56 days up to 168 days.

### 2.9. Sensory Analysis

Sensory acceptance and purchase intent of both UJJ and SJJ were performed at the beginning and at the end of the storage (168 days) at 25 °C. All the consumers reported at least once a week fruit juice consumption.

Juices were presented at ~10 °C in plastic cups coded with three-digits numbers and offered monadically in balanced order. Overall impression, aroma, color, flavor and viscosity were evaluated by consumers using a 9-points structured hedonic scale. Consumers were also asked to report the presence of any residual taste. The purchase intent was evaluated using a 5-points structured scale.

The study was approved by the ethics committee of the Federal University of Rio de Janeiro (approval number: 2.425.898).

### 2.10. Statistical Analysis

Data are expressed as mean ± standard deviation. Differences in phenolic compounds profile between UJJ and SJJ at each storage time were evaluated by unpaired Student’s *t*-test. Differences in chemical composition between storage times for UJJ were evaluated by one-way ANOVA followed by Dunnett’s post hoc test. Pearson correlation analysis was performed between instrumental color and anthocyanins content. The effects of sucrose addition and of storage on the sensory acceptance and purchase intent scores were investigated using Wilcoxon matched-pairs signed rank test and Mann–Whitney test, respectively. Statistical analyses were performed using GraphPad Prism software version 8.0 (San Diego, CA, USA) and results were considered significant when *p* < 0.05.

## 3. Results and Discussion

### 3.1. Sugars, Organic Acids and Phenolic Compounds Profiles and Sensory Acceptance of Steam Extracted Jaboticaba Juice

UJJ contained glucose, fructose and sucrose, and oxalic, tartaric, malic and citric acids (Table 1). The two monosaccharides accounted for 94% of the total sugars content, while citric acid was responsible for 91% of the organic acid content, in accordance with fruit [2] and juice data [17]. Ascorbic acid, which has already been reported in the fruit [2], was not observed in the present study, suggesting its degradation during steam extraction. These compounds have a direct influence on juice sweetness and acidity, thus contributing to juice sensory properties.

The main fruit phenolic compounds were investigated. No relevant differences were observed between UJJ and SJJ phenolic compounds profile (Table 2). Cyanidin-3-O-glucoside (C3G) was the most abundant phenolic compound in juices, accounting for ~41% of the total phenolics content, followed by gallic (~28%) and ellagic acids (~19%), similarly to fruit profile [2,18]. Upon comparison with a depulped jaboticaba juice [4], which had 22- and 101-times lower phenolic compounds and C3G contents, respectively, we can conclude that steam extraction was able to lixiviate phenolic compounds from the fruit, especially the anthocyanins from the peel. 

**Figure 1:**
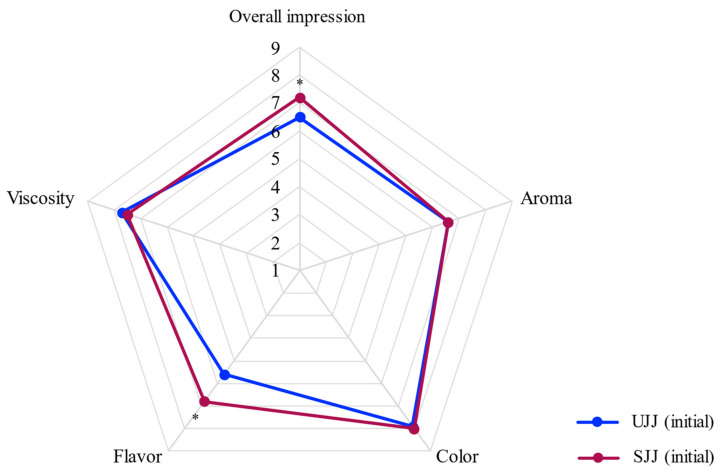
Average scores of descriptive sensory analysis of steam extracted jaboticaba juices. UJJ = unsweetened jaboticaba juice; SJJ = sweetened jaboticaba juice. Asterisks indicate means significantly different (Wilcoxon paired test; *p* < 0.05). Nine-point scale: 1 = dislike extremely; 2 = dislike very much; 3 = dislike moderately; 4 = dislike slightly; 5 = neither like nor dislike; 6 = like slightly; 7 = like moderately; 8 = like very much; 9 = like extremely.

In addition to yielding a juice rich in phenolics, the lixiviation of anthocyanins to the juice had a positive impact on its sensory acceptance, as color was one of the sensorial attributes with the highest scores in UJJ (Figure 1). In fact, jaboticaba depulped juice had lower appearance scores than our steam extracted juice probably due to its pale brownish pink color [4]. Moreover, upon addition of a natural colorant rich in anthocyanins to the depulped juice higher acceptance scores were obtained [19]. Flavor was the attribute with the lowest scores for UJJ, probably due to a residual bitter taste and an astringent mouthfeel, related to the presence of polyphenols [20], which was reported by about half of participants. Upon sucrose addition, flavor and overall impression scores increased from 5.6 ± 2.1 to 6.8 ± 1.6 and 6.5 ± 1.6 to 7.2 ± 1.4, respectively, and only a third of participants reported residual tastes for SJJ. Additionally, purchase intent scores increased from 2.9 ± 1.2 to 3.5 ± 0.9. In fact, it is known that sucrose addition attenuates the perception of bitterness and astringency, improving polyphenol-rich foods/beverages acceptability [20]. Additionally, people tend to prefer sweet-tasting foods and beverages [21]. Among the participants of our study, half informed to sweeten fresh fruit juices with sweeteners.

### 3.2. Sugars and Organic Acid Stabilities, Phenolic Compounds Degradation and Instrumental Color Modification during Storage of Unsweetened Jaboticaba Juice

Except for sucrose, sugar contents of juice remained stable during the whole storage time at 25 °C (Table 1). Among organic acids, tartaric acid was the only that showed decrease (14%), although of little relevance (Table 1). In general, sugars and organic acids are not susceptible to changes during storage at room temperature. The stability of these compounds is possibly associated to benefits in terms of sensorial and microbiological stabilities of juices.

**Figure 2:**
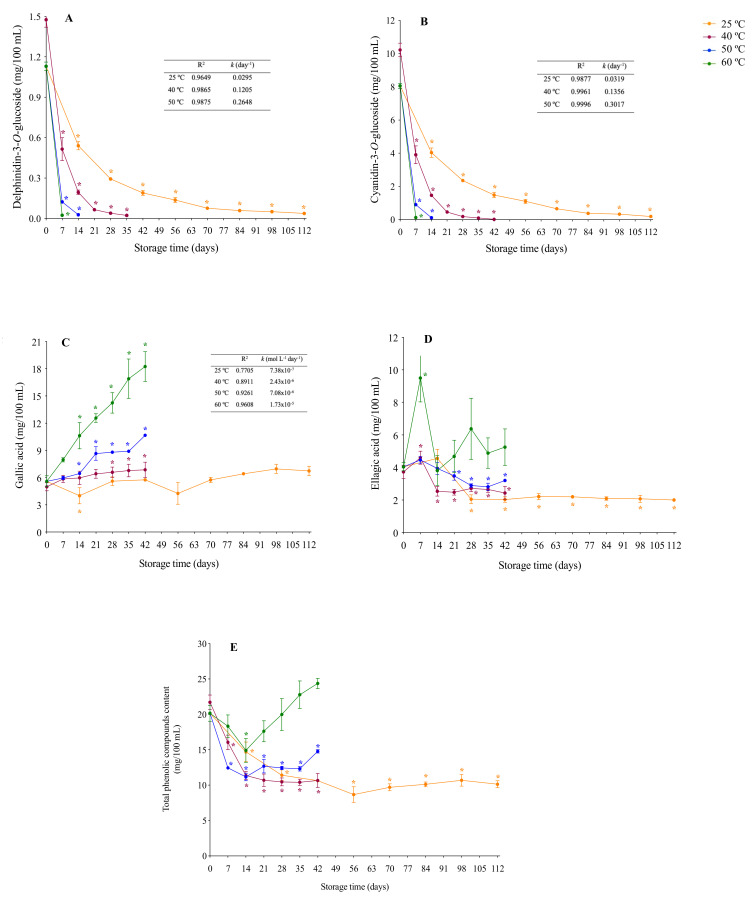
Contents of delphinidin-3-*O*-glucoside (D3G) (**A**), cyanidin-3-*O*-glucoside (C3G) (**B**), gallic acid (**C**), ellagic acid (**D**) and total phenolic compounds (**E**) of unsweetened jaboticaba juice during storage. First-order degradation kinetics parameters of D3G (**A**) and C3G (**B**) and zero-order formation kinetics parameters of gallic acid (**C**) are shown in the inserted tables. Asterisks indicate means significantly different from day zero for each temperature (One-way ANOVA followed by Dunnett’s post hoc test; *p* < 0.05).

Considering that phenolic compounds profile during storage were similar in UJJ and SJJ, henceforth only data from UJJ will be presented and discussed. An expressive decrease in anthocyanins contents was observed (Figure 2A,B), with C3G and D3G presenting similar losses (~98%) after 112 days of storage at 25 °C. The degradation of these compounds was expected since anthocyanins are known to be highly unstable and susceptible to degradation at room temperature [22]. At higher storage temperatures, anthocyanins degradation was even faster (e.g., total loss after 14 days at 60 °C). Extensive losses have also been reported in berries juices [3,4,11]. Although gallic acid content did not change after 112 days at 25 °C, it increased (from 38% to 226%) when juice was stored at higher temperatures (Figure 2C). Ellagic acid contents decreased from 50% (25 °C) to 21% (50 °C), while a 135% increase was observed after 7 days of storage at 60 °C and decreased thereafter (Figure 2D). These compounds may be formed from the hydrolysis of ellagitannins, which have been reported in jaboticaba [18,23,24]. However, castalagin and vescalagin, ellagitannins of lower polymerization degrees, were investigated but not identified. Thus, the observed increase in gallic and ellagic acids are probably due to the hydrolysis of tannins with higher polymerization degrees. However, these compounds were not analyzed, being a limitation of the present study. Overall, total phenolics contents decreased from 27% (50 °C) to 50% (25 °C) (Figure 2E), mainly driven by the reduction in C3G content. At 60 °C, no change was observed after 42 days of storage once the degradation of C3G was counterbalanced by the formation of gallic acid (Figure 2E). Considering the potential bioactivity of jaboticaba phenolic compounds, strategies to improve their stability during storage, such as addition of natural conservatives and/or cold storage, could be employed.

The loss of anthocyanins during storage directly affected juice instrumental color (r = –0.8963, *p* < 0.0001). At 25 °C, Δ*E* was distinguishable (1.5 < Δ*E* < 5) to the human eye [25] after 14 days of storage (Δ*E* = 4.0), became evident (Δ*E* > 5) after 42 days (Δ*E* = 5.7) and progressively increased thereafter (up to Δ*E* = 8.0) (Figure S2). Color change was mainly related to decreases in a* (11.73) and b* (3.92) values, of 67% and 55% respectively. Correlations were observed between total anthocyanins content (sum of D3G and C3G contents) and a* values (r = 0.9122, *p* < 0.0001) and b* values (r = 0.8837, *p* < 0.0001) values. Nevertheless, the juice still presented its purplish color even after 112 days of storage, which may be explained by different rates between anthocyanins degradation and color fading [26]. At higher temperatures, Δ*E* changed more rapidly. At 40 °C, evident color changes were observed after 21 days after storage (Δ*E* = 6.2) whereas at 50 and 60 °C, as soon as 7 days of storage (Δ*E* = 5.7 and 7.0, respectively) (Figure S2).

### 3.3. Sensory Acceptance and Microbiological Quality of Sweetened and Unsweetened Jaboticaba Juices after Six Months of Storage

**Figure 3:**
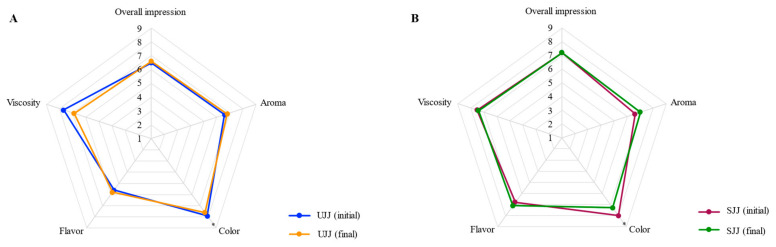
Average scores of descriptive sensory analysis of unsweetened (**A**) and sweetened (**B**) jaboticaba juices, UJJ and SJJ, respectively. Asterisks indicate means significantly different (Mann–Whitney unpaired test; *p* < 0.05). Nine-point scale: 1 = dislike extremely; 2 = dislike very much; 3 = dislike moderately; 4 = dislike slightly; 5 = neither like nor dislike; 6 = like slightly; 7 = like moderately; 8 = like very much; 9 = like extremely.

Storage of jaboticaba juices for 168 days at 25 °C did not affect their sensory acceptance and purchase intent, with the exception of color, which scores slightly decreased (~6%) (Figure 3). Therefore, even though anthocyanins were almost completely degraded and instrumental color showed evident changes, consumers acceptance regarding juice color remained high and would probably not impair the product global acceptance and commercial potential.

Although fruit juices are prone to spoilage, especially in long-term storage, steam extraction yielded jaboticaba juices free of thermotolerant coliforms and *Salmonella* sp. and with low numbers of possibly spoilage microorganisms (up to 90 colony-forming units per mL of juice) as heterotrophic bacteria, lactic acid bacteria, yeasts and molds. The extraction process was also effective to ensure adequate juice microbiological quality during storage. No growth of thermotolerant coliforms and *Salmonella* spp. was observed during 168 days of storage and presence of heterotrophic bacteria, lactic acid bacteria, yeasts and molds was limited to less than 10 colony-forming units per mL of juice for each of them (Table S1). Lopes et al. [11] observed that grape juice produced by the same method was microbiologically stable for 24 months. 

### 3.4. Degradation and Formation of Phenolic Compounds of Jaboticaba Juice during Storage

Considering the importance of phenolic compounds to jaboticaba juice sensory acceptance and potential health benefits, and that these compounds were either extremely degraded (anthocyanins) or formed (gallic acid) during storage, we performed kinetic studies, in order to investigate their transformation pathways.

**Figure 4:**
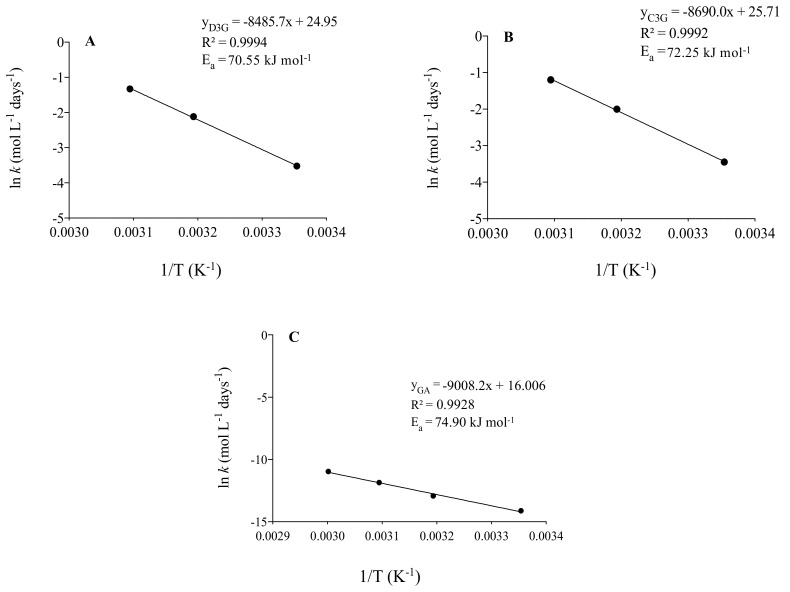
Arrhenius plots of delphinidin-3-*O*-glucoside (D3G) (**A**) and cyanidin-3-*O*-glucoside (C3G) (**B**) degradation and of gallic acid (**C**) formation.

Anthocyanins thermal degradation showed an exponential behavior, following a first-order reaction model (Figure 2A,B). R^2^ values (>0.96) confirmed that the Arrhenius model fitted the experimental data at temperatures ranging from 25 to 50 °C. Degradation rates constants (*k*) increased with temperature and results are in accordance with previously published data [27,28]. At each given temperature, C3G *k* value was higher than that of D3G, indicating that the former was degraded faster than the latter. Consequently, the half-life times for D3G and C3G degradation at 25 °C were 23.9 days and 21.7 days, respectively. Together, these results indicate that D3G is more stable than C3G in jaboticaba juice, which is the opposite behavior observed for these anthocyanins isolated from grapes [29], suggesting that the food matrix may affect the relationship between the structure of anthocyanins and their stability. From a thermodynamical point of view, the experimental activation energy values (E_a_) obtained from the Arrhenius plot (Figure 4A,B) for D3G (E_a_ = 70.55 kJ mol^−1^) and C3G (E_a_ = 72.25 kJ mol^−1^) suggest that the reaction rate of the latter is more sensitive to an increase in temperature than the former. E_a_ values are similar to that reported for total anthocyanins (E_a_ = 72.2 kJ mol^−1^) in black carrot juice [30]. While gallic acid contents remained constant when juice was stored at 25 °C, at higher temperatures (40, 50 and 60 °C) a linear increase was observed over time (R^2^ > 0.89). Gallic acid formation followed a zero-order reaction model and showed increasing formation rates (*k*) with temperature. The 7-fold increase between formation rate constants at 40 °C (*k* = 2.43 × 10^−6^ mol L^−1^ day^−1^) and 60 °C (*k* = 1.73 × 10^−5^ mol L^−1^ day^−1^) (Figure 2C) suggests a strong influence of temperature on the formation of this compound. The experimental activation energy for gallic acid formation was 74.90 kJ mol^−1^ (Figure 4C) and, to the best of our knowledge, our study reports it for the first time. Gallic acid is known as a degradation product of delphinidin, a compound that presents low thermal stability [28]. Additionally, gallic acid may be released by the hydrolysis of gallotannins during heating [31]. We can suppose that for jaboticaba juice the second reaction would be more relevant, once D3G contents were relatively low and therefore its degradation (~3 μmol/100 mL at 60 °C) would not be enough to cause the observed increase in gallic acid (~74 μmol/100 mL at 60 °C). Moreover, jaboticaba has been described as a fruit rich in gallotannins [24], a class of molecules that may be depolymerized yielding several gallic acid units.

**Figure 5:**
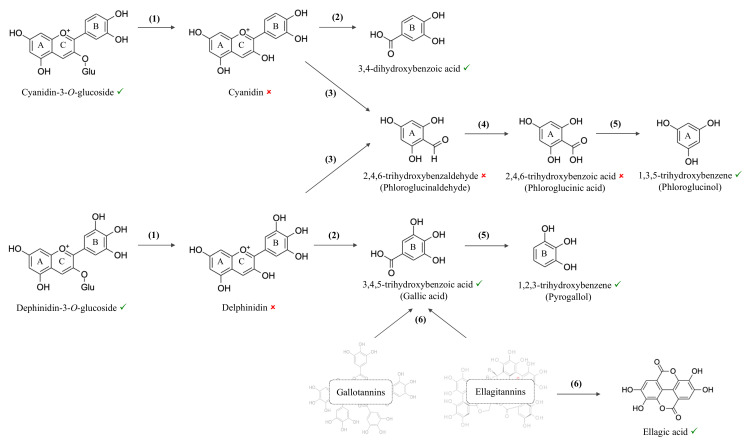
Proposed reaction pathways of phenolic compounds from jaboticaba juice during storage. Anthocyanins may undergo deglycosilation (reaction 1), followed by ring cleavage (reactions 2 and 3), oxidation (reaction 4) and decarboxylation (reaction 5), while tannins may undergo hydrolysis/depolymerization (reaction 6). Compounds marked with ✓ and 🗶 were observed and not observed in the juice, respectively.

The scheme in Figure 5 illustrates possible routes, precursors and products, considering the main phenolics identified, but not necessarily quantified, due to chromatographic limitations, in the jaboticaba juice during storage at all temperatures and previous literature data [28,31,32,33]. C3G and D3G may undergo deglycosylation forming the corresponding aglycones (reaction 1). Cyanidin and delphinidin, in their turn, may undergo ring cleavage (reactions 2 and 3) forming a phenolic acid arising from the B-ring (3,4-dihydroxybenzoic and gallic acids, respectively) and an aldehyde from the A-ring (2,4,6-trihydroxybenzaldehyde). In this study, we observed 3,4-dihydroxybenzoic and gallic acids, but not cyanidin and delphinidin, suggesting that these aglycones were transient species in anthocyanins degradation pathway. 2,4,6-trihydroxybenzaldehyde was also not observed and neither was its oxidation (reaction 4) product (2,4,6-trihydroxybenzoic acid), whereas 1,3,5-trihydroxybenzene, which may be formed from its decarboxylation (reaction 5), was observed. These results suggest that the aldehyde and its oxidation product are also transient species in this pathway. 1,2,3-trihydroxybenzene, the decarboxylation (reaction 5) product of gallic acid was also observed. In addition to anthocyanins degradation, hydrolysis/depolymerization (reaction 6) of gallotannins and ellagitannins seems to have taken place, yielding gallic and ellagic acids.

## 4. Conclusions

Jaboticaba juice produced by steam extraction incorporated the phenolic compounds from the fruit and showed adequate microbiological quality and good sensorial acceptance requiring neither the employment of any additional preservation technology nor the use of chemical preservatives. While sugars and organic acids contents were not modified during juice storage at 25 °C for 6 months, phenolics profile and contents changed, mainly because anthocyanins were almost completely degraded. Nevertheless, sensorial acceptance was not affected. Finally, steam extraction seems to be a viable method to produce jaboticaba juice with commercial potential, even though scale-up and marketing studies are still needed for this product to reach the market.

## Figures and Tables

**Table 1:** Sugars and organic acids contents of unsweetened jaboticaba juice stored at 25 °C for 112 days.

	Storage Time (Days)		
	0	56	112
Fructose (g/100 mL)	1.79 ± 0.11	1.74 ± 0.08	1.80 ± 0.09
Glucose (g/100 mL)	1.10 ± 0.11	1.02 ± 0.05	1.15 ± 0.07
Sucrose (g/100 mL)	0.2017 ± 0.0344	0.1732 ± 0.005	0.1267 ± 0.0001 *
Total sugars (g/100 mL)	3.09 ± 0.13	2.93 ± 0.09	3.08 ± 0.15
Oxalic acid (mg/100 mL)	22.30 ± 2.43	22.49 ± 2.54	23.00 ± 2.32
Tartaric acid (mg/100 mL)	20.87 ± 0.62	19.98 ± 0.73	17.94 ± 0.64 *
Malic acid (mg/100 mL)	92.53 ± 7.22	93.31 ± 3.80	93.76 ± 4.78
Citric acid (g/100 mL)	1.31 ± 0.07	1.32 ± 0.05	1.39 ± 0.04
Total organic acids (g/100 mL)	1.44 ± 0.08	1.46 ± 0.05	1.53 ± 0.04

Results are expressed as mean ± standard deviation of three process replicates. * indicate means significantly different from day zero (One-way ANOVA followed by Dunnett post hoc test; *F* = 6.825 and *p* = 0.0285 for sucrose; *F* = 39.31 and *p* = 0.00097 for tartaric acid).

**Table 2:** Phenolic compounds contents (mg/100 mL) of unsweetened and sweetened jaboticaba juices.

Phenolic Compound	Unsweetened	Sweetened
Cyanidin-3-*O*-glucoside	8.06 ± 0.15	7.91 ± 0.43
Delphinidin-3-*O*-glucoside	1.13 ± 0.03	1.10 ±0.06
Gallic acid	5.59 ± 0.61	5.24 ± 0.13
Myricetin-3-*O*-rhamnoside	0.48 ± 0.10	0.51 ± 0.03
Quercetin-3-*O*-rutinoside	0.71 ± 0.07	0.60 ± 0.01
Ellagic acid	3.91 ± 0.26	3.40 ± 0.12 *
Quercetin	traces	traces
*Trans*-cinnamic acid	0.132 ± 0.025	0.115 ± 0.003
Total	20.03 ± 1.13	18.89 ± 0.44

Results are expressed as mean ± standard deviations of three process replicates. * indicates mean significantly different from unsweetened jaboticaba juice (unpaired *t*-test; *p* = 0.0360).

## Data Availability

The datasets generated for this study are available on request to the corresponding author.

## Supplementary Materials

The following are available online at https://www.mdpi.com/article/10.3390/foods10040732/s1, Figure S1: The steam extractor, or steam juicer, is composed of three pieces, arranged one above the other: a water pan, a juice-collecting container and a fruit container. The water pan (i) is placed on a heat source in order to boil the water and, thus, to produce the steam for the process. The juice-collecting container (ii) has a central conical opening in its bottom, which allows the passage of the steam, and an outlet with a drain (iii), through which the juice is bottled. There is a clamp at the end of the drain which helps juice bottling. The fruit container (iv) is a basket with perforations, allowing the steam to reach the fruit and the juice to be drained, while keeping the fruit out of the reach of the extracted liquid. Figure S2: Changes in instrumental color of unsweetened jaboticaba juice during storage at 25 °C, 40 °C, 50 °C and 60 °C. Color components L*, a* and b* showed differences between initial and final storage time (one-way ANOVA followed by Dunnett’s post hoc test; *p* < 0.05). Table S1: Microbiological analysis of unsweetened and sweetened jaboticaba juices stored at 25 °C for 168 days ^a^.
