# Supplementary material for: Chemical, Microbiological and Sensory Stability of Steam Extracted Jaboticaba (Myrciaria jaboticaba) Juice
Source: Foods. 2021 Mar 30;10(4):732. doi: 10.3390/foods10040732 (PMC8065780; doi:10.3390/foods10040732)
Supplement: Supplementary file 1 [file foods-10-00732-s001.pdf]

Supplementary File:

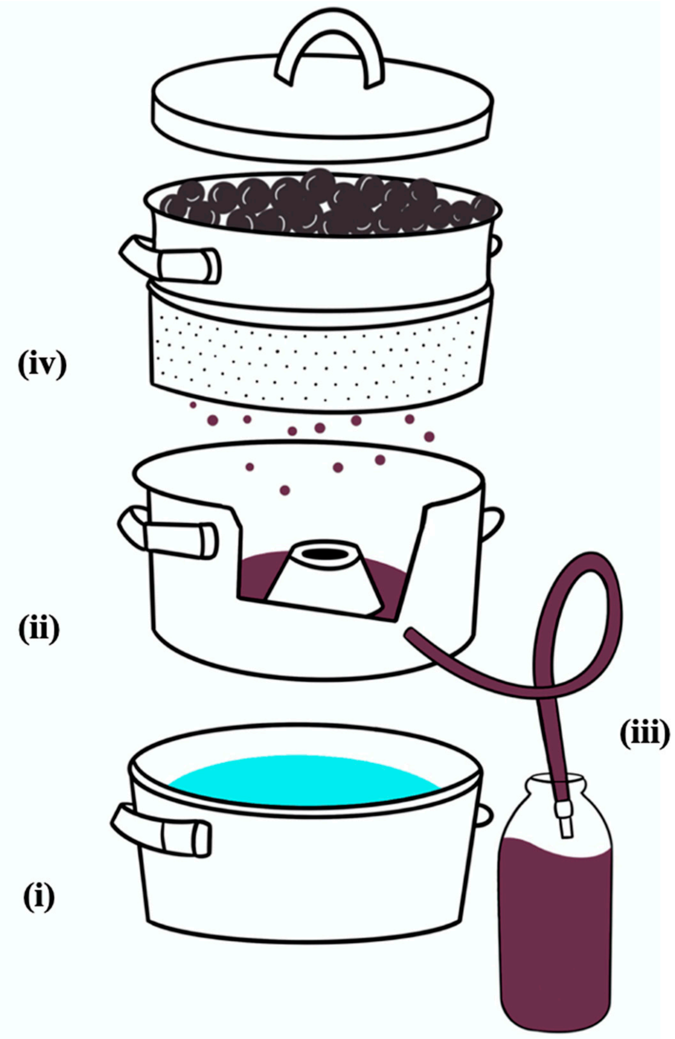

Figure S1. The steam extractor, or steam juicer, is composed of three pieces, arranged one above the other: a water pan, a juice-collecting container and a fruit container. The water pan (**i**) is placed on a heat source in order to boil the water and, thus, to produce the steam for the process. The juice-collecting container (**ii**) has a central conical opening in its bottom, which allows the passage of the steam, and an outlet with a drain (**iii**), through which the juice is bottled. There is a clamp at the end of the drain which helps juice bottling. The fruit container (**iv**) is a basket with perforations, allowing the steam to reach the fruit and the juice to be drained, while keeping the fruit out of the reach of the extracted liquid.

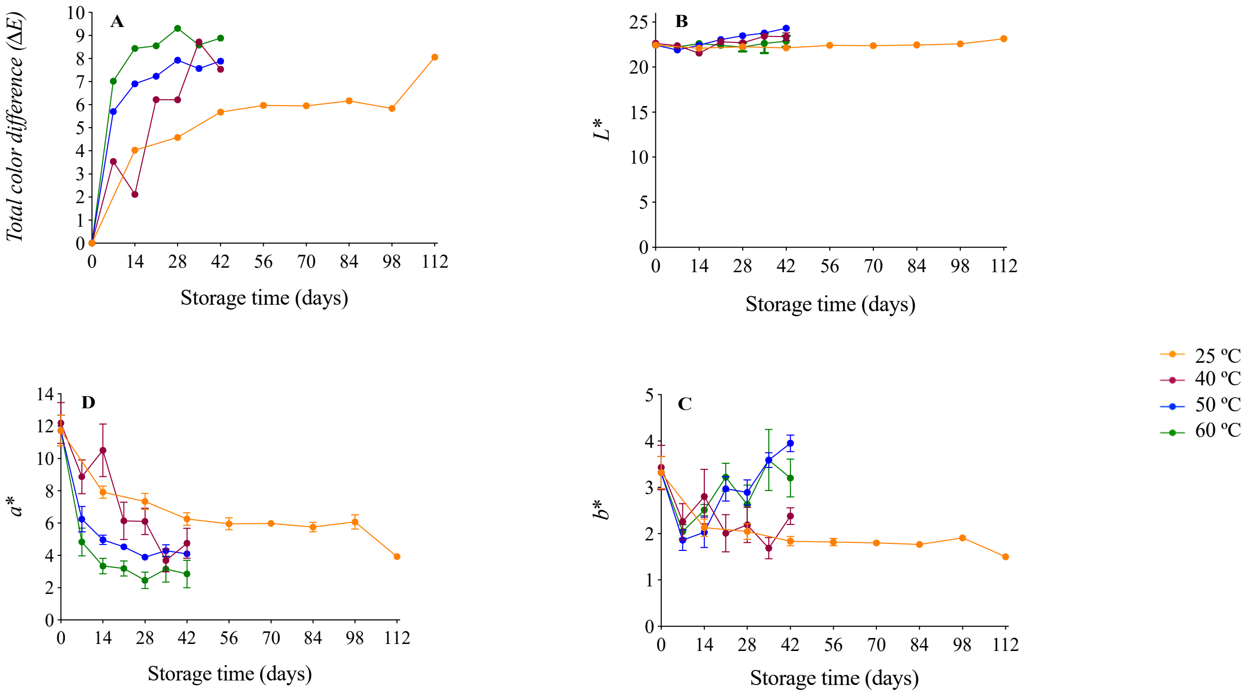

Figure S2. Changes in instrumental color of unsweetened jaboticaba juice during storage at 25 ºC, 40 ºC, 50 ºC and 60 ºC. Color components *L**, *a** and *b** showed differences between initial and final storage time (One-way ANOVA followed by Dunnett's post hoc test; $p < 0.05$).

Table S1. Microbiological analysis of unsweetened and sweetened jaboticaba juices stored at 25 ºC for 168 days[a].

Microorganism	Storage time (days)			
	0	56	112	168
Thermotolerant coliforms (MPN[b]/50 mL)	Absence	Absence	Absence	Absence
Salmonella sp. (25 mL)	Absence	Absence	Absence	Absence
Heterotrophic bacteria (CFU[c]/mL)	20	< 1.0	< 10	< 10
Yeasts and molds (CFU/mL)	90	< 1.0	< 10	< 10
Lactic acid bacteria (CFU/mL)	< 10	< 1.0	< 10	< 10

[a] Results for both juices were the same. [b]Most probable number. [c]Colony-forming units.
